# Supplementary material for: HDBind: encoding of molecular structure with hyperdimensional binary representations
Source: Sci Rep. 2024 Nov 23;14:29025. doi: 10.1038/s41598-024-80009-w (PMC11584749; doi:10.1038/s41598-024-80009-w)
Supplement: Supplementary file 1 — Supplementary Information. [file 41598_2024_80009_MOESM1_ESM.pdf]

Supplementary Information

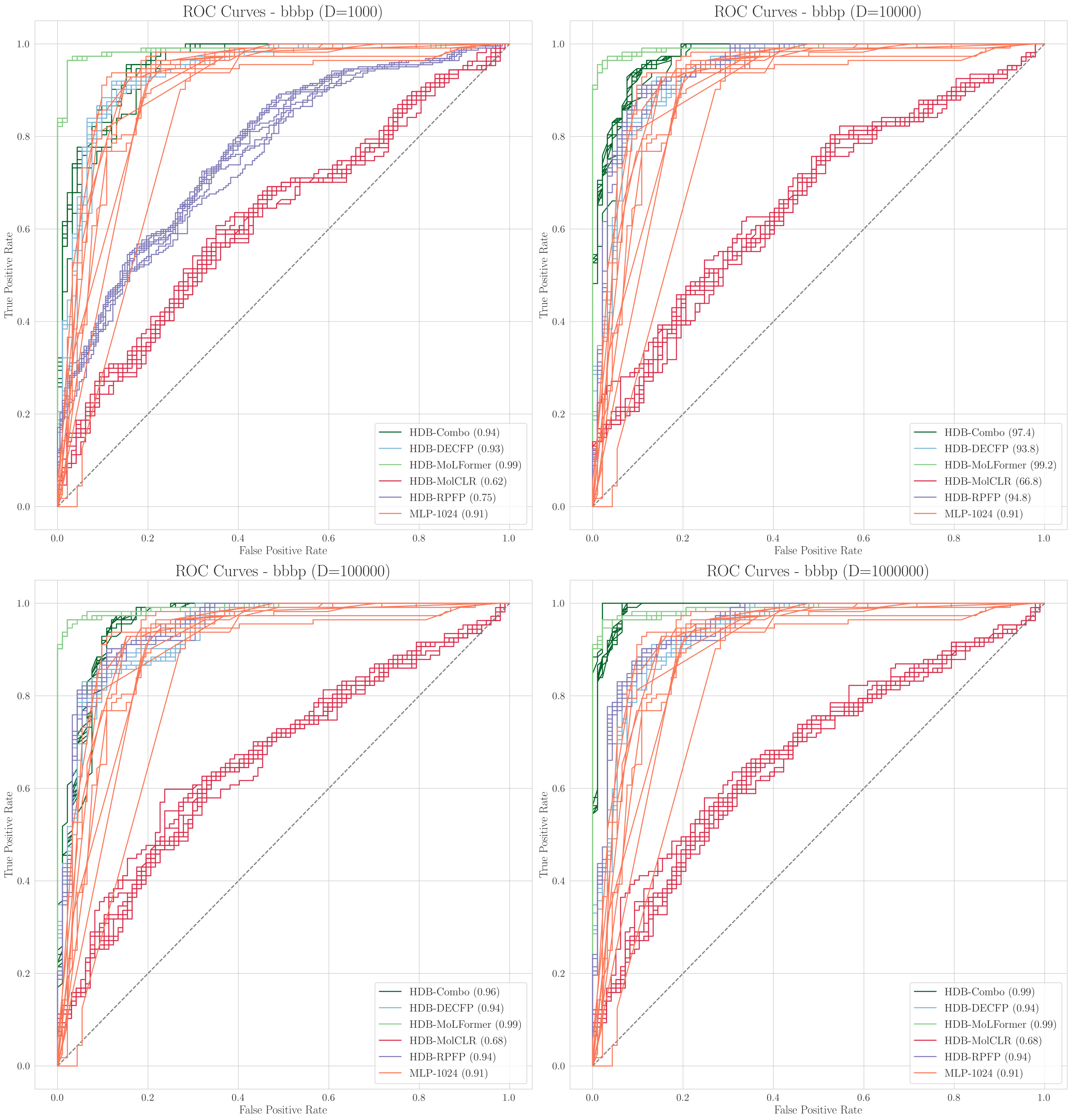

Figure 1. ROC Curves for the BBBP dataset. Values inside of '()' denote the mean ROC-AUC score, measured over 10 trials.

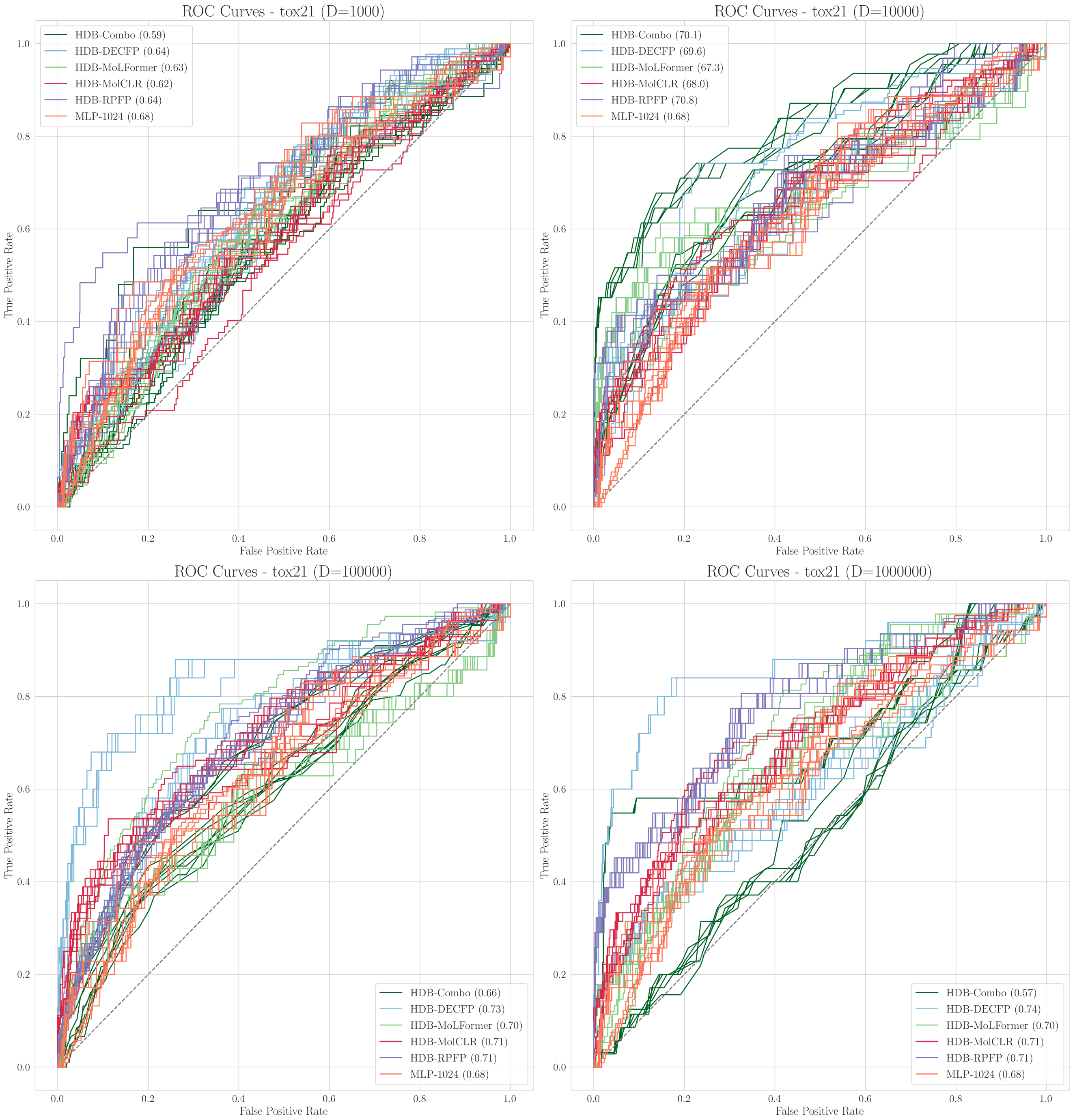

Figure 2. ROC Curves for the Tox21 dataset. Values inside of ‘()’ denote the mean ROC-AUC score, measured over 10 trials.

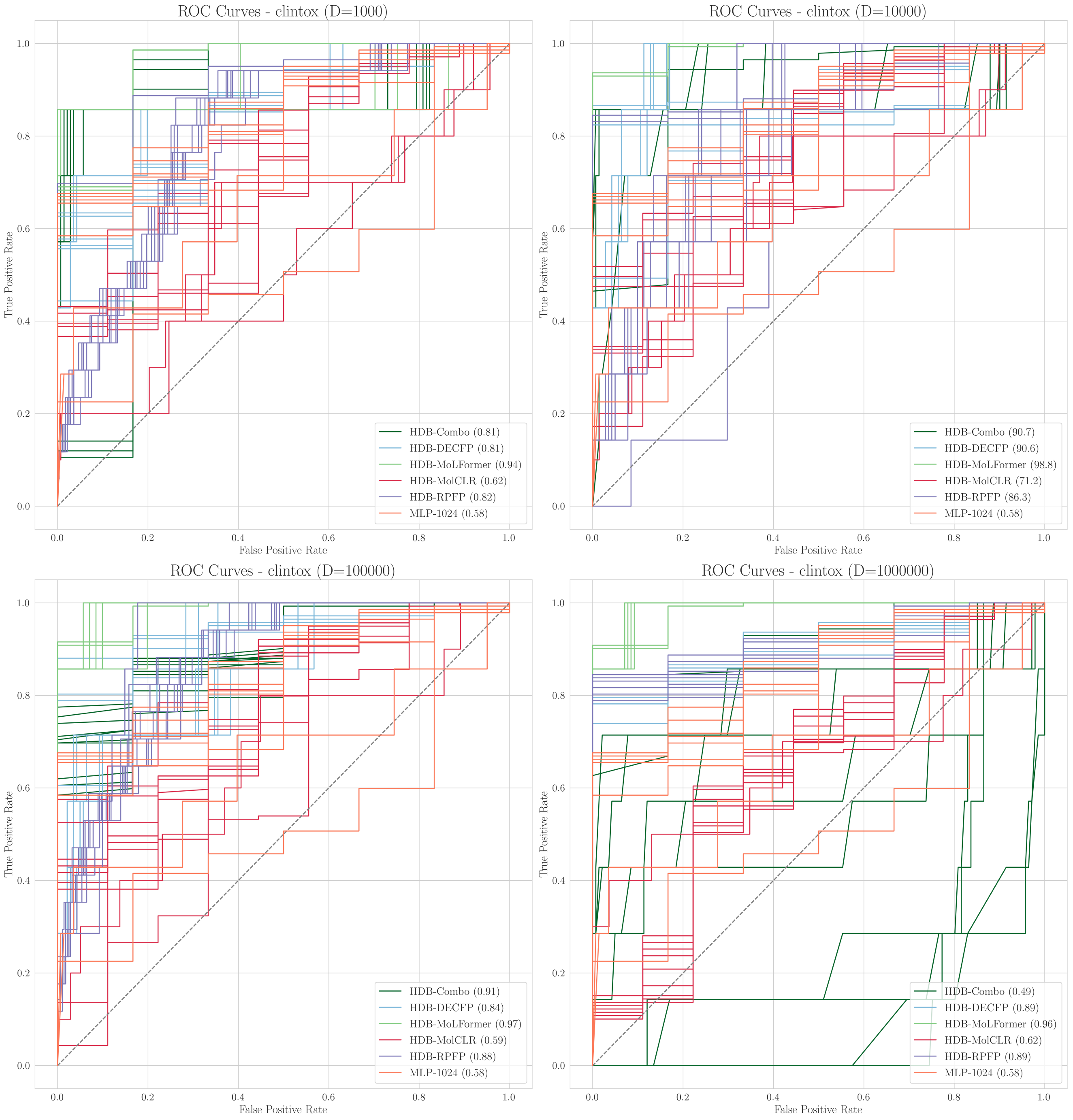

Figure 3. ROC Curves for the Clintox dataset. Values inside of '()' denote the mean ROC-AUC score, measured over 10 trials.

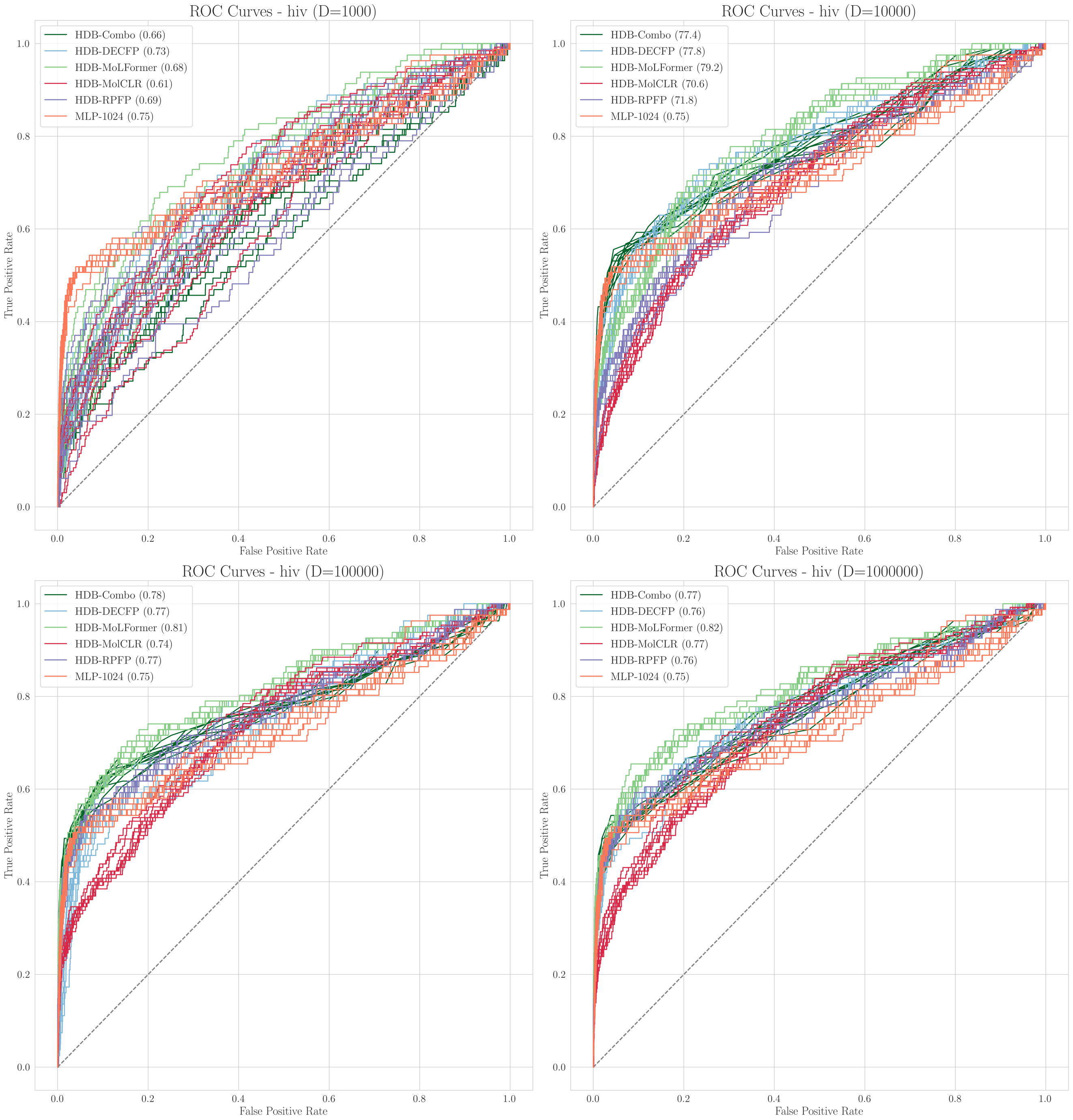

Figure 4. ROC Curves for the HIV dataset. Values inside of '()' denote the mean ROC-AUC score, measured over 10 trials.

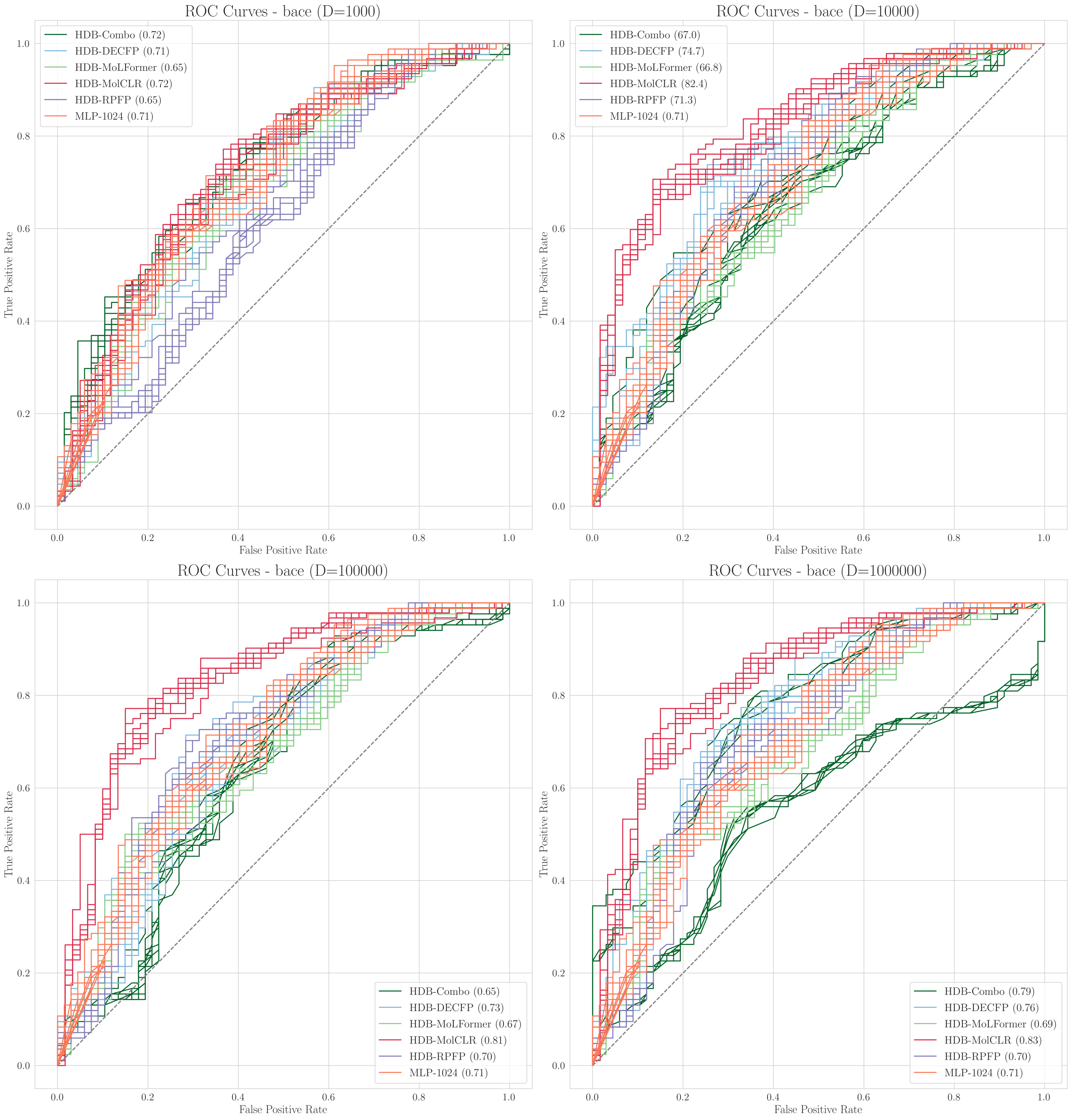

Figure 5. ROC Curves for the Bace dataset. Values inside of '()' denote the mean ROC-AUC score, measured over 10 trials.

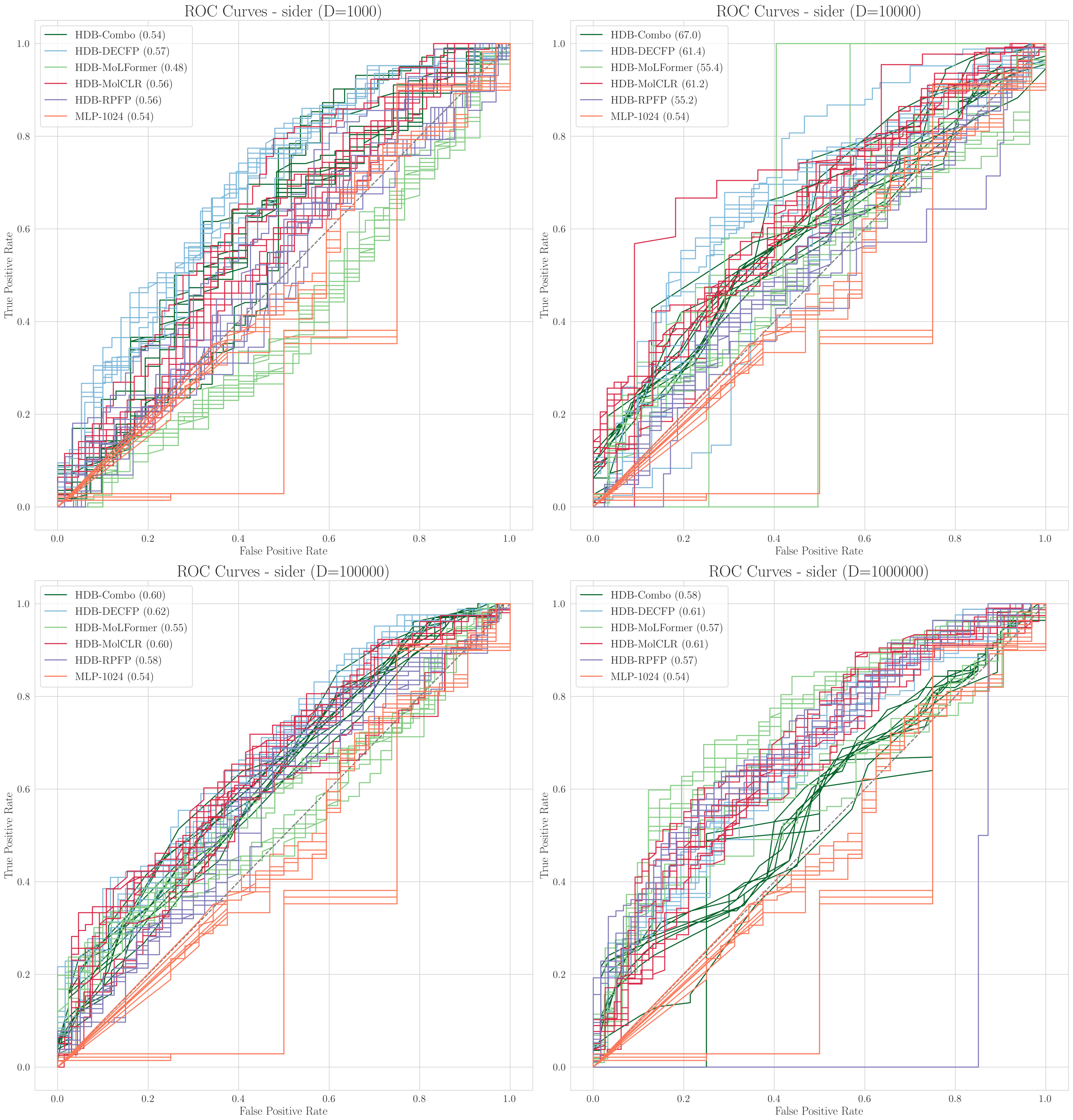

Figure 6. ROC Curves for the SIDER dataset. Values inside of '()' denote the mean ROC-AUC score, measured over 10 trials.

Method	BBBP	Tox21	ClinTox	HIV	BACE	SIDER
**Molecules**	2,039	7,831	1,478	41,127	1,513	1,427
**Tasks**	1	12	2	1	1	27
HDB-DECFP (1k-1)	93.6 (0.1)	65.8 (1.6)	87.4 (3.9)	73.4 (2.3)	68.0 (1.8)	57.4 (2.0)
HDB-DECFP (1k-2)	93.7 (0.2)	67.4 (1.1)	87.6 (2.6)	72.1 (2.1)	70.9 (1.2)	55.9 (2.0)
HDB-DECFP (1k-4)	94.2 (0.5)	63.4 (1.4)	81.9 (1.8)	68.9 (2.3)	68.6 (0.8)	60.4 (2.1)
HDB-DECFP (1024-1)	92.5 (0.5)	66.3 (1.0)	85.0 (4.7)	69.2 (2.2)	68.6 (2.4)	57.3 (1.9)
HDB-DECFP (1024-2)	93.7 (0.4)	72.4 (1.0)	79.2 (3.4)	68.9 (2.2)	74.1 (0.4)	55.3 (1.5)
HDB-DECFP (1024-4)	92.1 (0.5)	65.3 (1.3)	66.4 (2.4)	69.4 (1.8)	71.6 (0.8)	56.4 (1.4)
HDB-DECFP (2048-1)	93.1 (0.3)	72.1 (0.8)	88.3 (4.6)	70.7 (1.3)	70.6 (2.3)	60.6 (1.5)
HDB-DECFP (2048-2)	**94.3** (0.2)	70.5 (0.7)	86.2 (4.4)	71.3 (1.3)	73.8 (1.3)	61.1 (2.0)
HDB-DECFP (2048-4)	94.0 (0.1)	73.3 (0.7)	74.6 (2.6)	65.8 (0.8)	72.6 (1.1)	58.5 (1.8)
HDB-DECFP (10k-1)	93.9 (0.2)	72.5 (0.9)	88.4 (4.8)	75.7 (1.0)	68.4 (1.4)	61.9 (1.6)
HDB-DECFP (10k-2)	93.8 (0.2)	69.6 (0.8)	90.6 (4.0)	77.8 (0.3)	74.7 (1.1)	61.4 (1.6)
HDB-DECFP (10k-4)	94.1 (0.2)	68.7 (1.1)	83.0 (3.6)	77.9 (0.2)	73.2 (1.1)	60.3 (1.3)
HDB-DECFP (100k-1)	93.8 (0.2)	76.1 (0.5)	89.3 (3.1)	75.3 (0.4)	68.9 (1.7)	61.8 (1.5)
HDB-DECFP (100k-2)	93.8 (0.2)	73.0 (1.0)	90.1 (1.4)	77.6 (0.4)	73.8 (1.1)	61.8 (1.2)
HDB-DECFP (100k-4)	94.2 (0.2)	73.8 (0.8)	85.6 (4.6)	77.9 (0.3)	73.8 (1.4)	59.5 (1.4)
HDB-DECFP (1m-1)	93.8 (0.2)	73.0 (0.8)	**93.8** (1.8)	75.8 (1.2)	69.0 (1.9)	59.0 (1.4)
HDB-DECFP (1m-2)	93.8 (0.1)	75.0 (0.9)	90.0 (0.8)	78.0 (0.3)	**74.8** (1.1)	**63.1** (1.0)
HDB-DECFP (1m-4)	**94.3** (0.2)	**79.5** (0.6)	86.7 (0.9)	**78.8** (0.3)	74.2 (1.6)	61.2 (1.3)

Table 1. Comparison of HDB-DECFP models with varied ECFP length and radius parameters on MoleculeNet classification benchmarks. The standard deviation generally decreases with smaller radius parameter values while performance tends to increase with larger length parameter values. For $D = 1,000,000$, the best model is achieved for each task, with an exception for BBBP where a value of $D = 2048$ and ecfp radius of 2 tie the respective model using $D = 1,000,000$ and an ecfp radius of 4.

Method	BBBP	Tox21	ClinTox	HIV	BACE	SIDER
**Molecules**	2,039	7,831	1,478	41,127	1,513	1,427
**Tasks**	1	12	2	1	1	27
HDB-MoLFormer (1k)	98.6 (0.0)	64.1 (1.3)	92.1 (0.9)	72.7 (3.0)	**68.6** (1.3)	48.1 (1.8)
HDB-MoLFormer (10k)	**99.2** (0.0)	67.3 (1.0)	**98.8** (0.0)	79.2 (0.6)	66.8 (0.4)	55.4 (1.9)
HDB-MoLFormer (100k)	99.1 (0.1)	71.3 (0.9)	98.4 (0.7)	81.2 (0.3)	68.2 (0.5)	56.5 (2.1)
HDB-MoLFormer (1m)	99.0 (0.1)	**71.5** (1.0)	98.4 (0.8)	**81.6** (0.3)	68.3 (0.4)	**58.1** (2.1)

Table 2. Comparison of HDB-MoLFormer models on MoleculeNet classification benchmarks with varied hypervector dimension size D. Generally, increased dimension bring about improved roc-auc scores.

Method	BBBP	Tox21	ClinTox	HIV	BACE	SIDER
**Molecules**	2,039	7,831	1,478	41,127	1,513	1,427
**Tasks**	1	12	2	1	1	27
HDB-Combo (1k)	95.7 (0.5)	60.4 (1.3)	86.2 (1.7)	64.0 (3.2)	**72.4** (0.3)	54.9 (1.9)
HDB-Combo (10k)	97.4 (0.3)	**70.1** (1.2)	90.7 (3.4)	77.4 (0.8)	**67.0** (2.7)	58.8 (2.8)
HDB-Combo (100k)	95.9 (0.4)	69.7 (1.5)	**91.3** (1.9)	**77.4** (0.3)	65.3 (0.6)	**59.5** (2.7)
HDB-Combo (1m)	**99.1** (0.3)	58.1 (0.9)	69.6 (16.5)	77.2 (0.8)	64.1 (10.5)	59.0 (2.1)

Table 3. Comparison of HDB-Combo models on MoleculeNet classification benchmarks with varied ECFP length and radius parameters. The standard deviation generally decreases with smaller radius parameter values while performance tends to increase with larger length parameter values.

Model	D	ER-1% (r)	ER-1% (a)
MoleHD*	10k	15.12 (1.65)	2.63 (0.70)
HDB-RPFP	10k	25.76 (2.59)	5.46 (1.87)
HDB-RPFP	100k	25.31 (2.90)	6.84 (1.43)
HDB-RPFP	1m	25.50 (2.51)	7.73 (1.81)
HDB-MolCLR	10k	24.55 (4.27)	7.99 (1.06)
HDB-MolCLR	100k	28.40 (3.93)	6.02 (1.36)
HDB-MolCLR	1m	28.44 (4.30)	6.91 (0.80)
HDB-MoLFormer	10k	28.24 (2.18)	5.72 (0.95)
HDB-MoLFormer	100k	35.81 (3.86)	8.15 (1.80)
HDB-MoLFormer	1m	**36.69** (3.43)	6.87 (0.70)
HDB-DECFP (r=1)	10k	28.19 (2.74)	6.74 (1.80)
HDB-DECFP (r=2)	10k	30.70 (3.07)	8.02 (1.06)
HDB-DECFP (r=4)	10k	32.15 (2.87)	7.85 (0.95)
HDB-DECFP (r=1)	100k	28.35 (3.67)	8.33 (1.59)
HDB-DECFP (r=2)	100k	32.28 (2.51)	6.75 (0.88)
HDB-DECFP (r=4)	100k	31.80 (2.68)	8.46 (1.22)
HDB-DECFP (r=1)	1m	29.32 (3.69)	8.81 (1.68)
HDB-DECFP (r=2)	1m	33.55 (2.34)	9.80 (1.24)
HDB-DECFP (r=4)	1m	34.83 (2.01)	10.46 (1.78)
HDB-Combo	10k	35.82 (5.24)	4.72 (0.97)
HDB-Combo	100k	32.03 (2.12)	**30.27** (2.10)
HDB-Combo	1m	16.49 (9.44)	17.84 (8.49)
MLP*	-	30.79 (5.04)	7.61 (1.84)
Pafnucy[1,2]	-	-	3.46 (1.97)
GRIM[2,3]	-	-	4.78 (3.11)

Table 4. LIT-PCBA roc-enrichment (ER-1%) factor metrics, averaged over 15 target datasets where each value is reported as the mean over 10 random seeds. 'r' denotes random split and 'a' denotes ave bias minimizing split provided by the authors[4]. Values correspond to the ER-1% metrics, averaged over all 15 targets using 10 random seeds for each. Values inside of the parentheses represent the standard deviation over the 10 random seeds for each model, averaged over the 15 protein targets. * denotes our implementation of the MoleHD and MLP baselines. Bold indicates best overall model and best HDC model. Values highlighted in green represent those that have statistically significant improvement in ER-1% upon the best previously reported method, GRIM[2,3]. Values highlighted in yellow represent those models for which the mean ER-1% is higher than the mean GRIM score. Values in red represent those that have mean ER-1% lower than GRIM[2,3].

Representation	#Parameters	Device	Type	Time (mol/s)	Molecules/day
ECFP[5]	-	IBM Power 9	CPU	10,000	864,000,000
MoLFormer[6]	46,781,184	Nvidia V100 (16GB)	GPU	692	59,828,514
MolCLR[7]	2,404,196	Nvidia V100 (16GB)	GPU	6,250	540,000,000

Table 5. Latency measurements for the different molecular feature extractors considered in this work. All measurement taken on a single Lassen compute node. A single GPU is used for MoLFormer and MolCLR extraction.

length	radius	Time (mol/s)	E (J/mol.)
1000	1	2628	0.062
1000	2	2301	0.070
1000	4	1844	0.088
10000	1	2333	0.069
10000	2	2072	0.078
10000	4	1704	0.096
100000	1	1024	0.159
100000	2	975	0.169
100000	4	888	0.185
1000000	1	155	1.059
1000000	2	155	1.060
1000000	4	153	1.079

Table 6. ECFP latency and energy analysis with respect to length and radius.

Model	Layers	Trainable parameters
MLP-small	(1024, 128), (128,2)	131,458
MLP-large	(1024, 512), (512, 256), (256,128),(128,2)	688,384

Table 7. Description of the representative large and small MLP baseline models. For each set of parentheses (i.e. layer), the first number denotes the input size and the second denotes the output size.

Model	Device	Encode (J/mol)	Test (J/mol) $\times 10^{-6}$	1	10	100	1000	10000	100000
HDB-DECFP	GPU	0.057	3.37	0.057	0.057	0.058	0.061	0.091	0.394
HDB-MoLFormer	GPU	0.389	3.37	0.389	0.389	0.389	0.392	0.422	0.725
HDB-Combo	GPU	0.496	3.37	0.496	0.496	0.496	0.499	0.529	0.832
MLP-small	GPU	0.071	2.30	0.071	0.071	0.071	0.073	0.094	0.300
MLP-large	GPU	0.071	9.18	0.071	0.071	0.072	0.080	0.163	0.989
HDB-DECFP	FPGA	0.057	.75	0.057	0.057	0.057	0.058	0.065	0.132
HDB-MoLFormer	FPGA	0.389	.75	0.389	0.389	0.389	0.390	0.396	0.464
HDB-Combo	FPGA	0.496	.75	0.496	0.496	0.496	0.497	0.503	0.571

Table 8. Energy profiling results for HDBind models and our smallest and largest MLP baselines, respectively MLP-small and MLP-large. All values correspond to CPU and GPU power terms provided by variorum[8]. All steps of feature extraction are included for encoding the input, such as extraction of the MoLFormer embedding itself as well as calculation of ECFPs. All HDC models use $D = 1$k to facilitate comparison to FPGA baseline, which is relatively constrained in memory resources to the CPU and GPU. Green denotes models for which screening a particular number of proteins can be done with less energy than our MLP-small baseline. Values in yellow correspond to those models for which at a particular point can be done with less energy than MLP-large but still require more energy than MLP-small. Values in red correspond to those models for which screening require more energy than either of the MLP-small or MLP-large baselines.

			roc-auc				er-1.0				Actives
			mean	std	min	max	mean	std	min	max	mean
model	D	target									
HDB-DECFP	100000	ADRB2	0.67	0.14	0.41	0.80	0.00	0.00	0.00	0.00	17.00
		ALDH1	0.71	0.04	0.65	0.76	10.99	3.90	4.99	15.18	5363.00
		FEN1	0.78	0.10	0.69	0.93	23.91	6.76	15.22	34.78	360.00
		GBA	0.73	0.06	0.67	0.82	14.47	4.34	4.88	21.95	163.00
		IDH1	0.74	0.03	0.67	0.80	9.63	3.84	0.00	11.11	39.00
		KAT2A	0.64	0.02	0.59	0.67	11.88	3.68	4.17	18.75	194.00
		MAPK1	0.66	0.03	0.60	0.70	3.64	1.94	0.00	9.09	308.00
		MTORC1	0.61	0.07	0.48	0.68	3.19	1.79	0.00	4.17	97.00
		OPRK1	0.46	0.10	0.35	0.61	0.00	0.00	0.00	0.00	24.00
		PKM2	0.67	0.07	0.60	0.77	7.89	3.30	1.47	13.24	546.00
		PPARG	0.72	0.03	0.68	0.79	11.11	15.98	0.00	33.33	24.00
		TP53	0.62	0.03	0.55	0.68	10.18	5.16	5.26	15.79	64.00
		VDR	0.73	0.05	0.67	0.80	10.44	1.16	7.88	12.73	655.00
	1000000	ADRB2	0.77	0.04	0.66	0.82	0.00	0.00	0.00	0.00	17.00
		ALDH1	0.71	0.04	0.64	0.76	11.10	4.16	4.61	15.55	5363.00
		FEN1	0.89	0.05	0.81	0.94	31.96	4.80	21.74	39.13	360.00
		GBA	0.73	0.08	0.57	0.82	16.34	5.21	7.32	24.39	163.00
		IDH1	0.77	0.04	0.70	0.83	15.19	8.99	0.00	22.22	39.00
		KAT2A	0.64	0.03	0.59	0.68	12.64	3.83	6.25	18.75	194.00
		MAPK1	0.67	0.02	0.64	0.70	4.68	1.69	2.60	10.39	308.00
		MTORC1	0.64	0.04	0.56	0.69	3.33	1.70	0.00	4.17	97.00
		OPRK1	0.52	0.01	0.49	0.54	0.00	0.00	0.00	0.00	24.00
		PKM2	0.71	0.07	0.60	0.78	8.55	3.05	1.47	13.24	546.00
		PPARG	0.70	0.01	0.68	0.73	9.44	8.40	0.00	16.67	24.00
		TP53	0.66	0.05	0.60	0.74	17.89	5.63	5.26	26.32	64.00
		VDR	0.78	0.04	0.71	0.81	12.10	1.78	8.48	14.55	655.00

Table 9. ROC-AUC and enrichment factor in true positives at a 1% false positive rate (er-1) metrics for HDB-DECFP across all 15 LIT-PCBA target sets.

			roc-auc				er-1.0				Actives
			mean	std	min	max	mean	std	min	max	mean
model	D	target									
HDB-RPFP	100000	ADRB2	0.56	0.01	0.55	0.58	0.00	0.00	0.00	0.00	17.00
		ALDH1	0.74	0.00	0.73	0.74	9.08	0.69	7.59	9.82	5363.00
		FEN1	0.82	0.01	0.80	0.83	17.17	1.68	15.22	19.57	360.00
		GBA	0.74	0.00	0.73	0.74	13.66	1.26	12.20	14.63	163.00
		IDH1	0.65	0.02	0.60	0.67	0.00	0.00	0.00	0.00	39.00
		KAT2A	0.65	0.01	0.64	0.66	8.75	2.15	6.25	12.50	194.00
		MAPK1	0.69	0.00	0.68	0.69	3.97	0.66	2.60	5.19	308.00
		MTORC1	0.55	0.02	0.51	0.58	2.08	2.20	0.00	4.17	97.00
		OPRK1	0.56	0.01	0.55	0.58	0.00	0.00	0.00	0.00	24.00
		PKM2	0.74	0.01	0.73	0.75	9.12	1.05	7.35	11.03	546.00
		PPARG	0.70	0.02	0.69	0.75	15.00	5.27	0.00	16.67	24.00
		TP53	0.67	0.03	0.62	0.70	12.63	3.68	5.26	15.79	64.00
		VDR	0.69	0.02	0.66	0.71	9.58	0.80	8.48	10.91	655.00
	1000000	ADRB2	0.59	0.01	0.57	0.60	0.00	0.00	0.00	0.00	17.00
		ALDH1	0.76	0.00	0.76	0.76	10.56	0.57	9.75	11.46	5363.00
		FEN1	0.80	0.01	0.78	0.81	15.33	1.08	13.04	16.30	360.00
		GBA	0.65	0.01	0.62	0.66	4.88	0.00	4.88	4.88	163.00
		IDH1	0.78	0.02	0.73	0.79	20.00	4.68	11.11	22.22	39.00
		KAT2A	0.67	0.00	0.66	0.67	10.21	0.66	8.33	10.42	194.00
		MAPK1	0.70	0.01	0.69	0.70	5.06	0.41	3.90	5.19	308.00
		MTORC1	0.64	0.01	0.62	0.66	0.00	0.00	0.00	0.00	97.00
		OPRK1	0.70	0.01	0.69	0.71	1.67	5.27	0.00	16.67	24.00
		PKM2	0.74	0.01	0.71	0.75	9.12	1.05	7.35	10.29	546.00
		PPARG	0.80	0.02	0.76	0.83	11.67	8.05	0.00	16.67	24.00
		TP53	0.72	0.02	0.68	0.74	14.74	2.22	10.53	15.79	64.00
		VDR	0.76	0.01	0.75	0.77	10.36	1.05	9.09	12.12	655.00

Table 10. ROC-AUC and enrichment factor in true positives at a 1% false positive rate (er-1) metrics for HDB-RPFP across all 15 LIT-PCBA target sets.

model	D	target	roc-auc mean	std	min	max	er-1.0 mean	std	min	max	Actives mean
HDB-MolCLR	100000	ADRB2	0.48	0.04	0.40	0.53	0.00	0.00	0.00	0.00	17.00
		ALDH1	0.72	0.01	0.70	0.72	8.47	0.59	7.07	9.15	5363.00
		FEN1	0.92	0.01	0.90	0.92	30.22	1.90	26.09	32.61	360.00
		GBA	0.78	0.01	0.77	0.80	10.24	2.24	7.32	14.63	163.00
		IDH1	0.77	0.01	0.76	0.78	1.11	3.51	0.00	11.11	39.00
		KAT2A	0.66	0.00	0.65	0.67	4.58	1.64	2.08	6.25	194.00
		MAPK1	0.71	0.01	0.71	0.72	6.75	1.02	5.19	9.09	308.00
		MTORC1	0.64	0.02	0.61	0.67	2.92	3.43	0.00	8.33	97.00
		OPRK1	0.63	0.02	0.61	0.66	0.00	0.00	0.00	0.00	24.00
		PKM2	0.70	0.00	0.69	0.70	2.35	0.90	1.47	3.68	546.00
		PPARG	0.58	0.01	0.57	0.58	0.00	0.00	0.00	0.00	24.00
		TP53	0.66	0.02	0.61	0.68	6.84	2.54	5.26	10.53	64.00
		VDR	0.78	0.01	0.77	0.79	10.36	0.60	9.70	10.91	655.00
	1000000	ADRB2	0.48	0.05	0.38	0.56	0.00	0.00	0.00	0.00	17.00
		ALDH1	0.73	0.00	0.72	0.74	8.49	0.56	7.43	9.22	5363.00
		FEN1	0.92	0.00	0.91	0.92	30.33	2.64	25.00	33.70	360.00
		GBA	0.78	0.01	0.77	0.79	11.71	1.92	9.76	14.63	163.00
		IDH1	0.74	0.01	0.71	0.75	11.11	0.00	11.11	11.11	39.00
		KAT2A	0.71	0.00	0.71	0.72	4.58	0.88	4.17	6.25	194.00
		MAPK1	0.69	0.00	0.68	0.70	6.75	0.82	5.19	7.79	308.00
		MTORC1	0.54	0.01	0.51	0.56	3.75	3.65	0.00	8.33	97.00
		OPRK1	0.67	0.01	0.66	0.69	0.00	0.00	0.00	0.00	24.00
		PKM2	0.70	0.00	0.69	0.71	3.68	0.60	2.94	4.41	546.00
		PPARG	0.56	0.00	0.55	0.56	0.00	0.00	0.00	0.00	24.00
		TP53	0.71	0.01	0.70	0.73	5.26	0.00	5.26	5.26	64.00
		VDR	0.77	0.01	0.76	0.79	10.06	0.96	9.09	12.12	655.00

Table 11. ROC-AUC and enrichment factor in true positives at a 1% false positive rate (er-1) metrics for HDB-MolCLR across all 15 LIT-PCBA target sets.

model	D	target	roc-auc				er-1.0				Actives
			mean	std	min	max	mean	std	min	max	mean
HDB-MoLFormer	100000	ADRB2	0.91	0.02	0.88	0.94	5.00	10.54	0.00	25.00	17.00
		ALDH1	0.69	0.00	0.68	0.69	4.38	0.17	4.17	4.61	5363.00
		FEN1	0.92	0.01	0.91	0.92	38.04	2.51	34.78	41.30	360.00
		GBA	0.82	0.01	0.80	0.83	14.63	2.82	7.32	17.07	163.00
		IDH1	0.86	0.01	0.83	0.87	10.00	3.51	0.00	11.11	39.00
		KAT2A	0.64	0.01	0.63	0.65	3.75	1.32	0.00	4.17	194.00
		MAPK1	0.70	0.00	0.69	0.71	6.23	1.19	3.90	7.79	308.00
		MTORC1	0.54	0.03	0.48	0.57	2.50	2.15	0.00	4.17	97.00
		OPRK1	0.76	0.03	0.70	0.80	16.67	0.00	16.67	16.67	24.00
		PKM2	0.76	0.00	0.75	0.76	6.76	0.76	5.15	7.35	546.00
		PPARG	0.58	0.03	0.53	0.61	0.00	0.00	0.00	0.00	24.00
		TP53	0.63	0.00	0.63	0.64	5.26	0.00	5.26	5.26	64.00
		VDR	0.73	0.01	0.72	0.75	5.45	0.76	3.64	6.06	655.00
	1000000	ADRB2	0.61	0.04	0.56	0.66	0.00	0.00	0.00	0.00	17.00
		ALDH1	0.72	0.00	0.72	0.72	6.29	0.22	5.95	6.62	5363.00
		FEN1	0.92	0.00	0.92	0.93	33.26	1.47	31.52	36.96	360.00
		GBA	0.81	0.01	0.79	0.82	18.54	1.71	17.07	21.95	163.00
		IDH1	0.89	0.01	0.87	0.90	11.11	0.00	11.11	11.11	39.00
		KAT2A	0.72	0.00	0.71	0.73	5.62	2.21	2.08	8.33	194.00
		MAPK1	0.69	0.00	0.69	0.69	5.45	0.55	5.19	6.49	308.00
		MTORC1	0.58	0.01	0.57	0.59	6.25	2.20	4.17	8.33	97.00
		OPRK1	0.84	0.01	0.83	0.85	0.00	0.00	0.00	0.00	24.00
		PKM2	0.71	0.01	0.70	0.72	3.16	1.04	0.74	4.41	546.00
		PPARG	0.55	0.00	0.55	0.56	0.00	0.00	0.00	0.00	24.00
		TP53	0.62	0.00	0.61	0.63	5.26	0.00	5.26	5.26	64.00
		VDR	0.71	0.02	0.68	0.74	4.06	1.14	3.03	6.67	655.00

Table 12. ROC-AUC and enrichment factor in true positives at a 1% false positive rate (er-1) metrics for HDB-MoLFormer across all 15 LIT-PCBA target sets.

model	D	target	roc-auc mean	roc-auc std	roc-auc min	roc-auc max	er-1.0 mean	er-1.0 std	er-1.0 min	er-1.0 max	Actives mean
HDB-Combo	100000	ADRB2	1.00	0.00	1.00	1.00	100.00	0.00	100.00	100.00	17.00
		ALDH1	0.53	0.01	0.51	0.55	1.53	0.30	1.14	2.11	5363.00
		FEN1	0.97	0.00	0.96	0.97	73.55	3.12	65.88	76.09	360.00
		GBA	0.73	0.02	0.68	0.74	35.09	5.37	24.39	39.02	163.00
		IDH1	0.44	0.05	0.36	0.52	0.75	2.37	0.00	7.50	39.00
		KAT2A	0.86	0.01	0.84	0.87	19.36	2.11	15.57	22.89	194.00
		MAPK1	0.97	0.00	0.97	0.97	60.71	2.76	57.14	66.23	308.00
		MTORC1	0.94	0.00	0.93	0.94	66.12	2.70	60.86	70.83	97.00
		OPRK1	0.92	0.01	0.90	0.93	16.67	0.00	16.67	16.67	24.00
		PKM2	0.93	0.02	0.90	0.95	60.96	9.60	42.70	72.63	546.00
		PPARG	0.69	0.04	0.62	0.74	0.00	0.00	0.00	0.00	24.00
		TP53	0.64	0.01	0.63	0.67	4.85	1.31	1.11	5.26	64.00
		VDR	0.57	0.01	0.56	0.58	2.12	0.63	1.21	3.27	655.00
	1000000	ADRB2	0.97	0.04	0.89	1.00	67.20	37.38	0.00	100.00	17.00
		ALDH1	0.54	0.02	0.53	0.56	1.16	0.18	0.82	1.34	5363.00
		FEN1	0.81	0.11	0.62	0.90	14.91	12.96	0.30	27.17	360.00
		GBA	0.69	0.10	0.55	0.79	19.44	16.02	0.00	36.59	163.00
		IDH1	0.48	0.05	0.41	0.56	0.11	0.36	0.00	1.13	39.00
		KAT2A	0.79	0.11	0.53	0.87	12.31	9.89	0.00	22.09	194.00
		MAPK1	0.86	0.09	0.69	0.94	20.64	11.46	2.49	40.86	308.00
		MTORC1	0.90	0.01	0.89	0.91	37.59	12.01	12.50	50.00	97.00
		OPRK1	0.68	0.20	0.39	0.94	13.64	7.25	0.00	19.73	24.00
		PKM2	0.94	0.02	0.92	0.96	58.95	6.47	50.74	65.62	546.00
		PPARG	0.64	0.11	0.45	0.81	0.00	0.00	0.00	0.00	24.00
		TP53	0.65	0.07	0.54	0.74	2.63	2.77	0.00	5.26	64.00
		VDR	0.56	0.08	0.48	0.64	2.03	0.45	1.08	2.42	655.00

Table 13. ROC-AUC and enrichment factor in true positives at a 1% false positive rate (er-1) metrics for HDB-Combo across all 15 LIT-PCBA target sets.

D	Single core (train)	Single core (test)	Max core (train)	Max core (test)
1000	2.3	2.0	2.2	4.5
10000	16.2	14.0	3.9	4.5
100000	118.2	97.7	19.5	23.0

Table 14. HDBind processing latency speedup on GPU versus CPU (single core, max cores) for training and testing. The advantage of the GPU grows with increasing dimension sizes D. We set the PyTorch thread count to 43, i.e. the number of physical cores - 1.

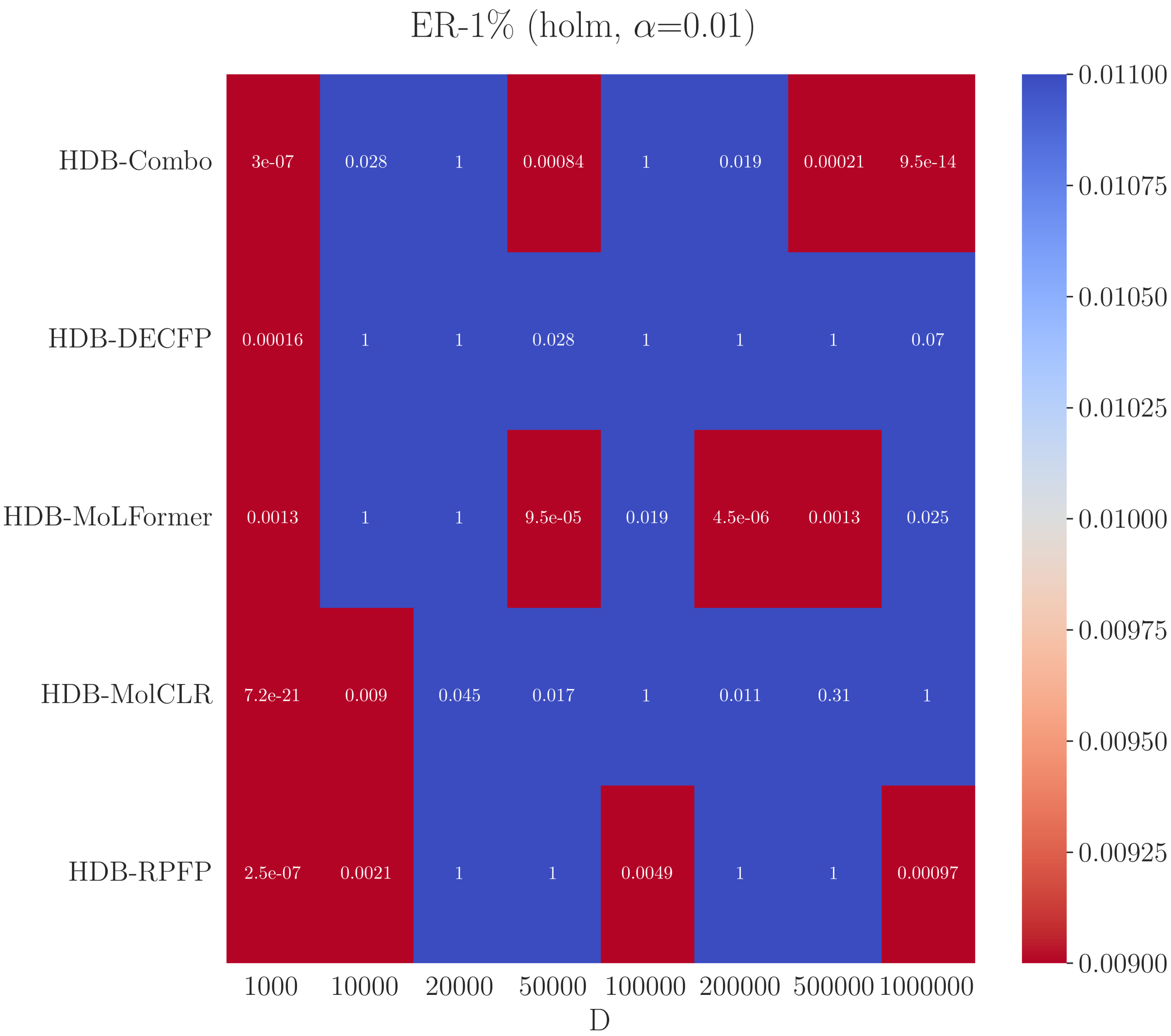

Figure 7. Paired t-test of HDBind models compared to MLP baseline models on the random split using the roc-auc metric distributions over all datasets (15 protein targets) and random seeds (10). Red cells indicate a statistically significant difference in the means of the respective roc-auc metric distributions (model, D) and blue cells indicate a non-significant difference. in The holm step-down correction is applied for multiple hypothesis testing and we use $\alpha = 0.01$.

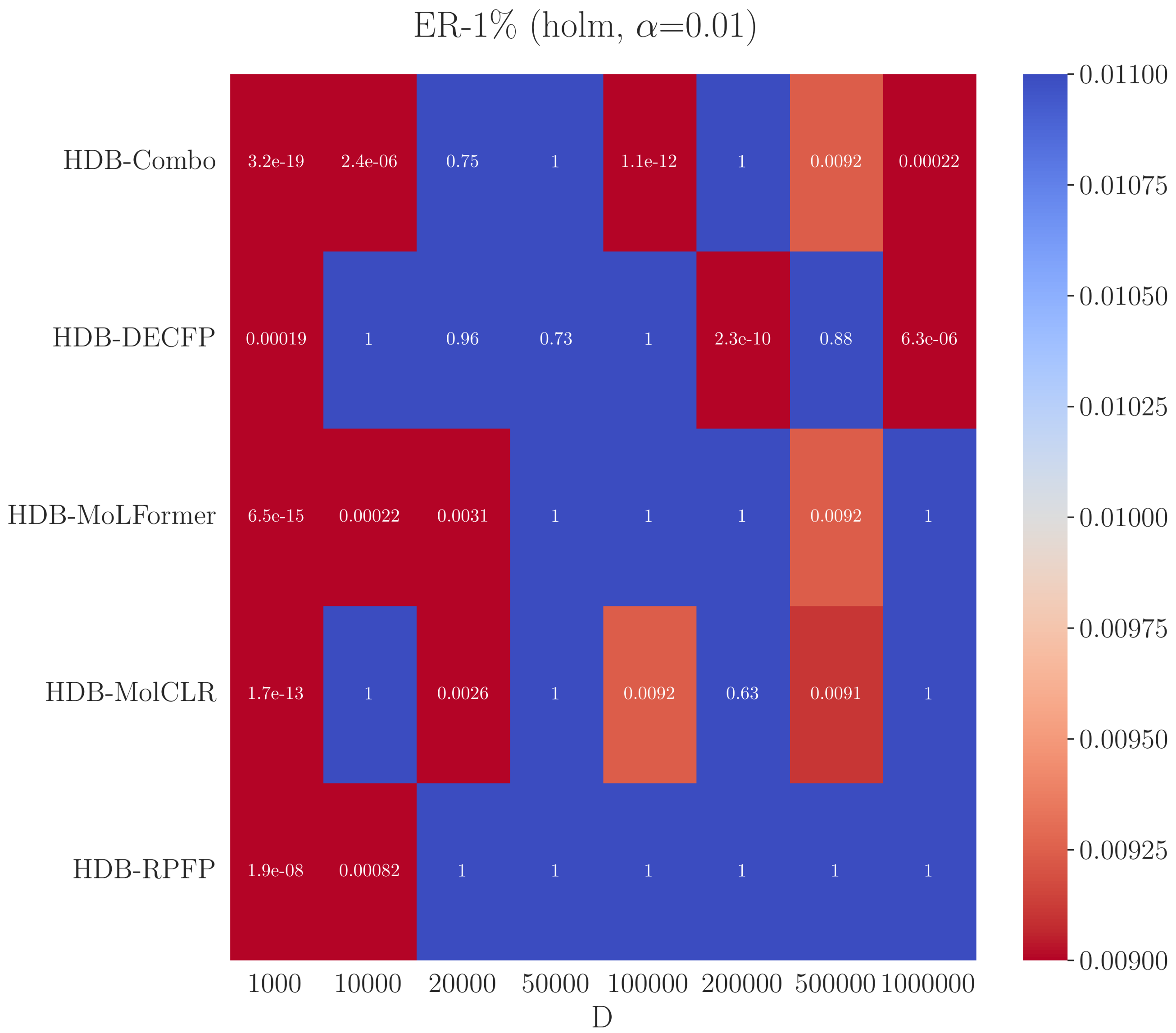

Figure 8. Paired t-test of HDBind models compared to MLP baseline models on the AVE bias-minimizing split using the roc-auc metric distributions over all datasets (15 protein targets) and random seeds (10). Red cells indicate a statistically significant difference in the means of the respective roc-auc metric distributions (model, D) and blue cells indicate a non-significant difference. in The holm step-down correction is applied for multiple hypothesis testing and we use $\alpha = 0.01$.

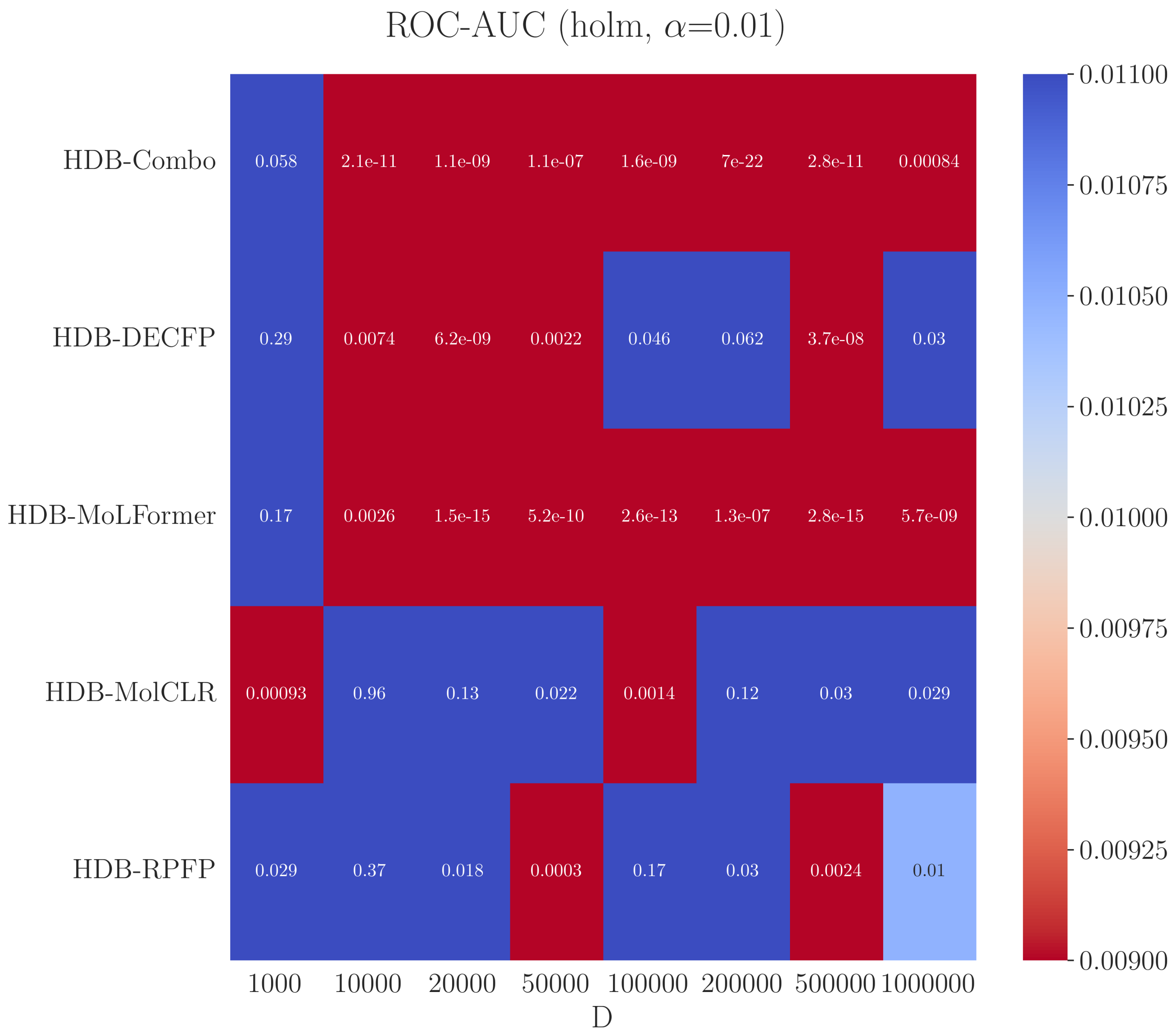

Figure 9. Paired t-test of HDBind models compared to MLP baseline models on the random split using the roc-auc metric distributions over all datasets (15 protein targets) and random seeds (10). Red cells indicate a statistically significant difference in the means of the respective roc-auc metric distributions (model, D) and blue cells indicate a non-significant difference. in The holm step-down correction is applied for multiple hypothesis testing and we use $\alpha = 0.01$.

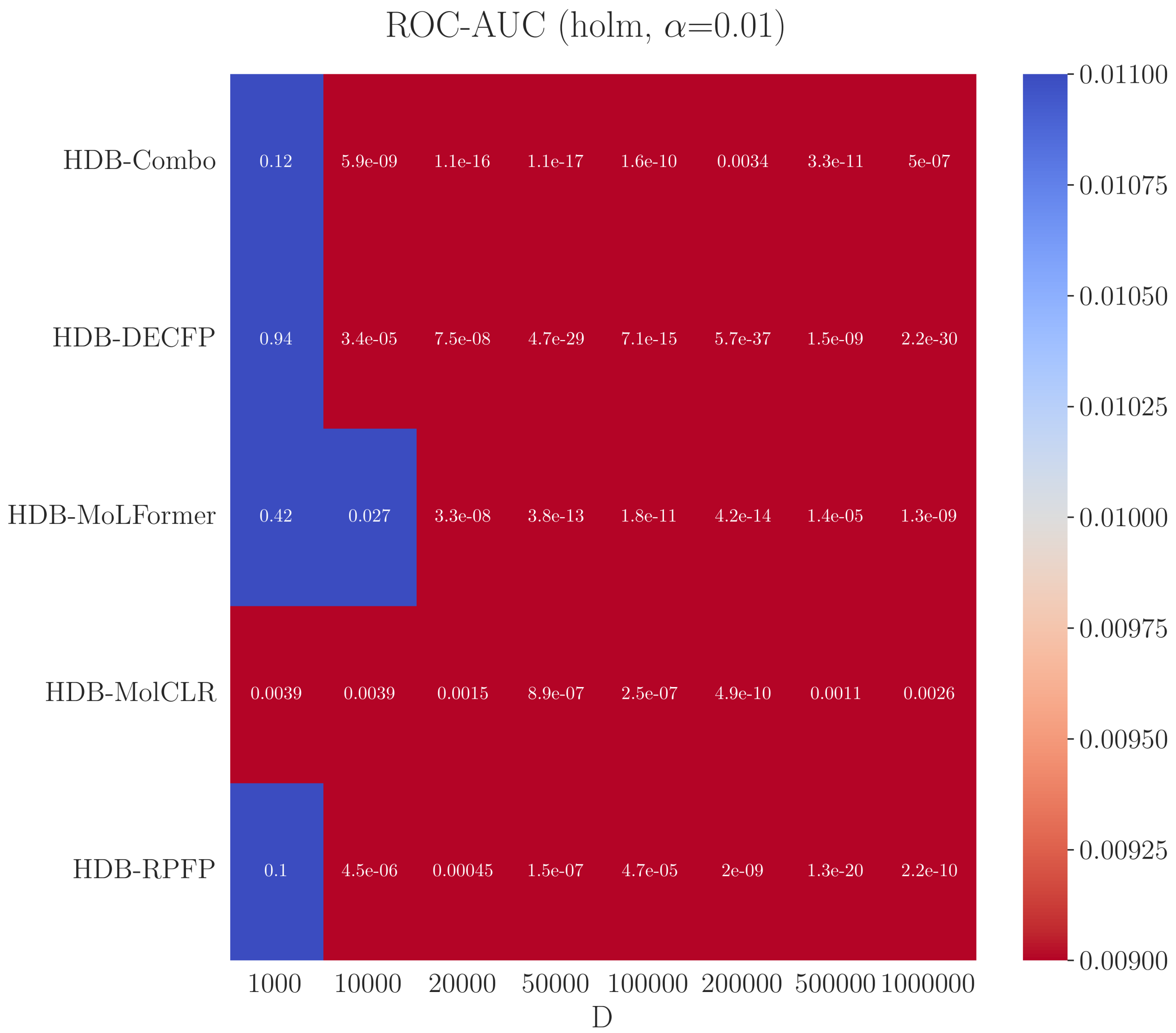

Figure 10. Paired t-test of HDBind models compared to MLP baseline models on the AVE bias-minimizing split using the roc-auc metric distributions over all datasets (15 protein targets) and random seeds (10). Red cells indicate a statistically significant difference in the means of the respective roc-auc metric distributions (model, D) and blue cells indicate a non-significant difference. in The holm step-down correction is applied for multiple hypothesis testing and we use $\alpha = 0.01$.

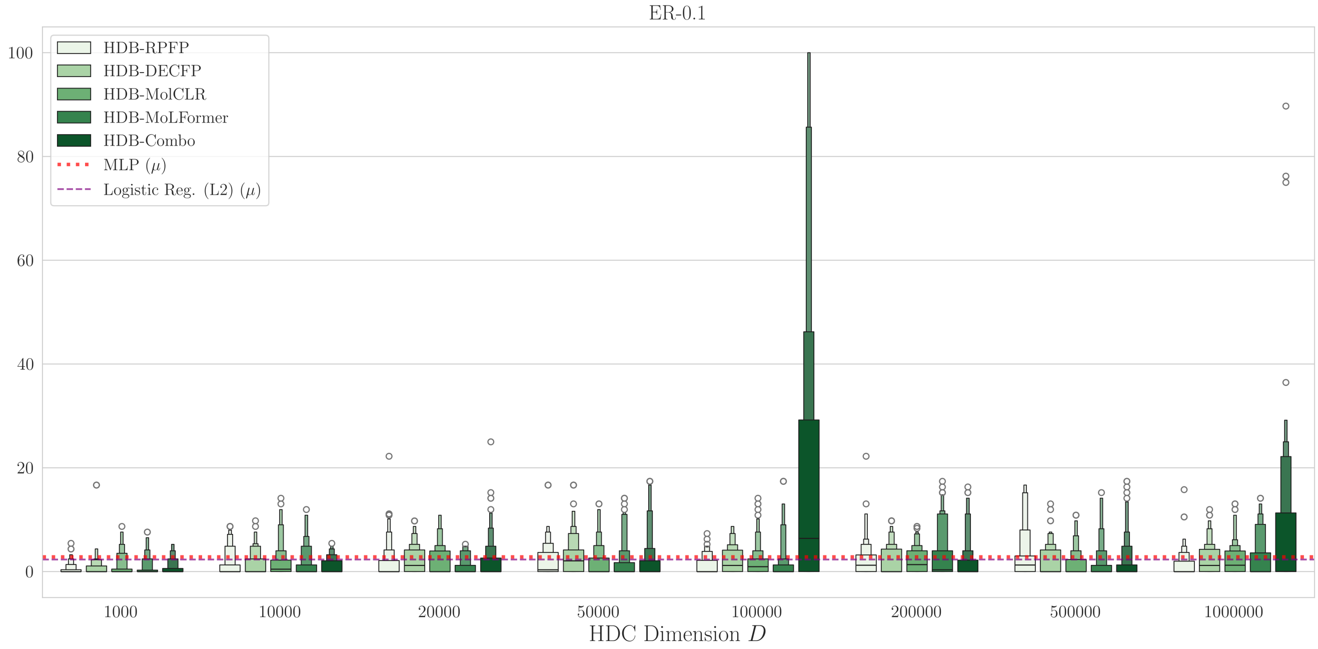

Figure 11. Distribution of the roc-enrichment with FPR=1/1000, compared to MLP and logistic regression baseline models.

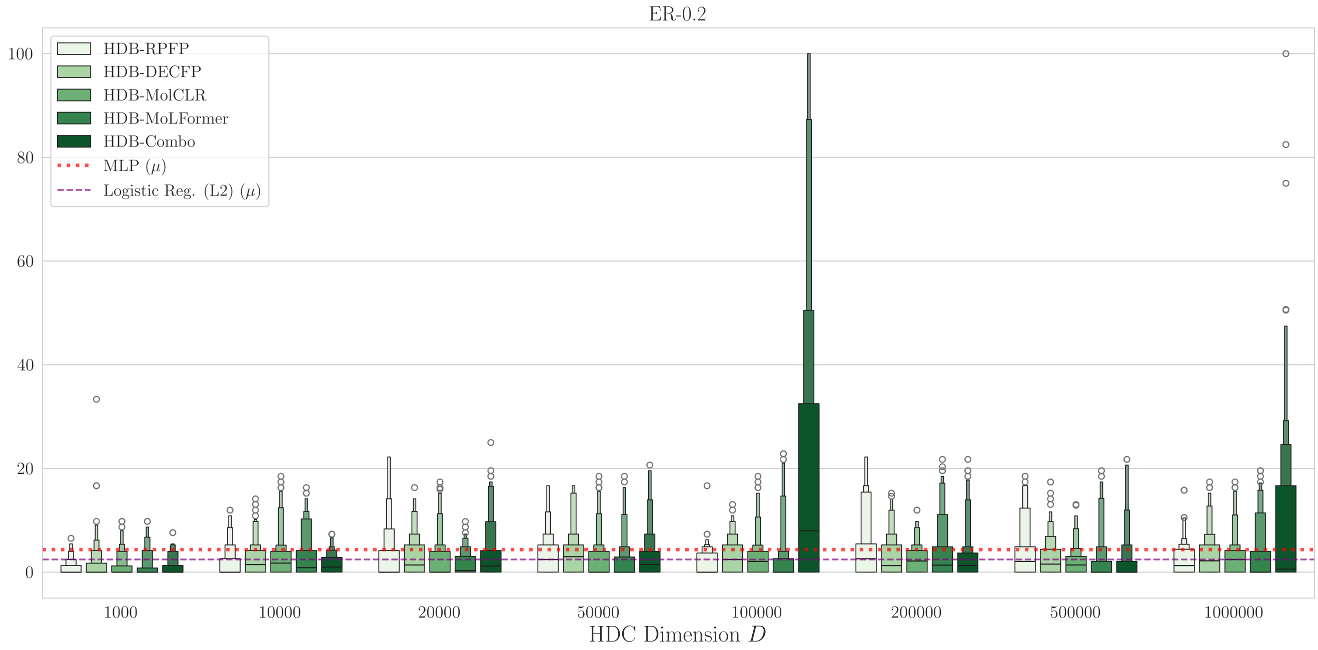

Figure 12. Distribution of the roc-enrichment with FPR=2/1000, compared to MLP and logistic regression baseline models.

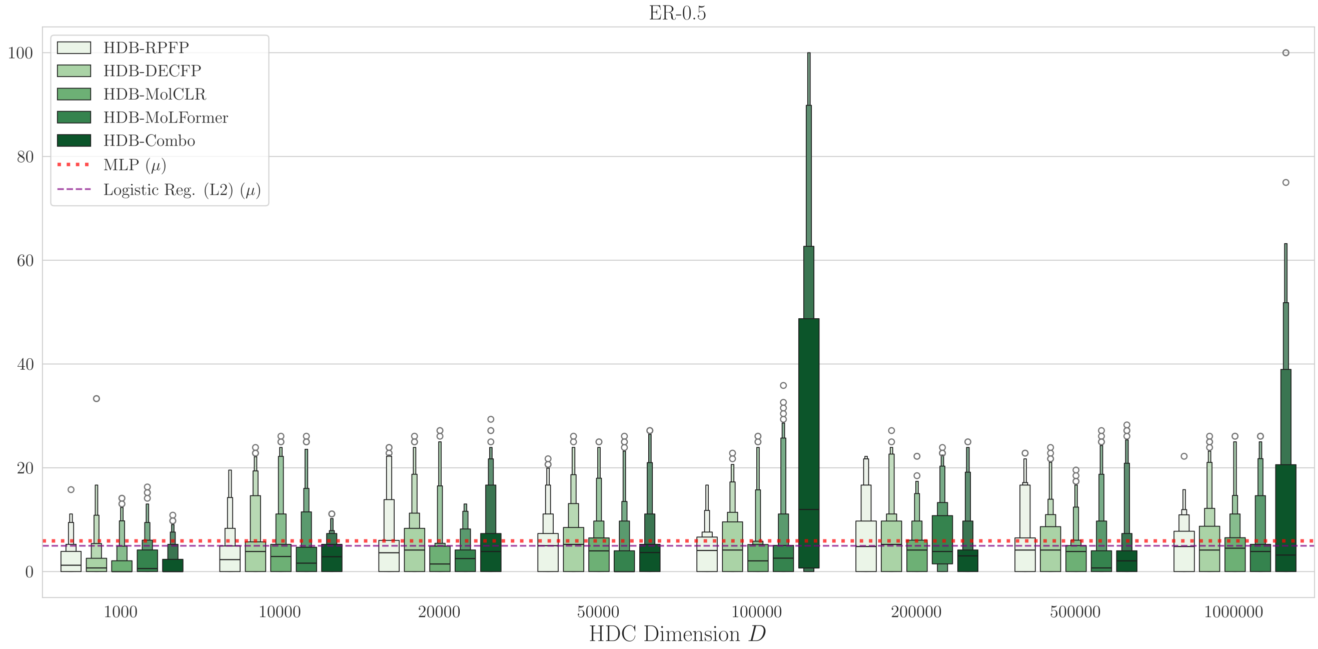

Figure 13. Distribution of the roc-enrichment with FPR=5/1000, compared to MLP and logistic regression baseline models.

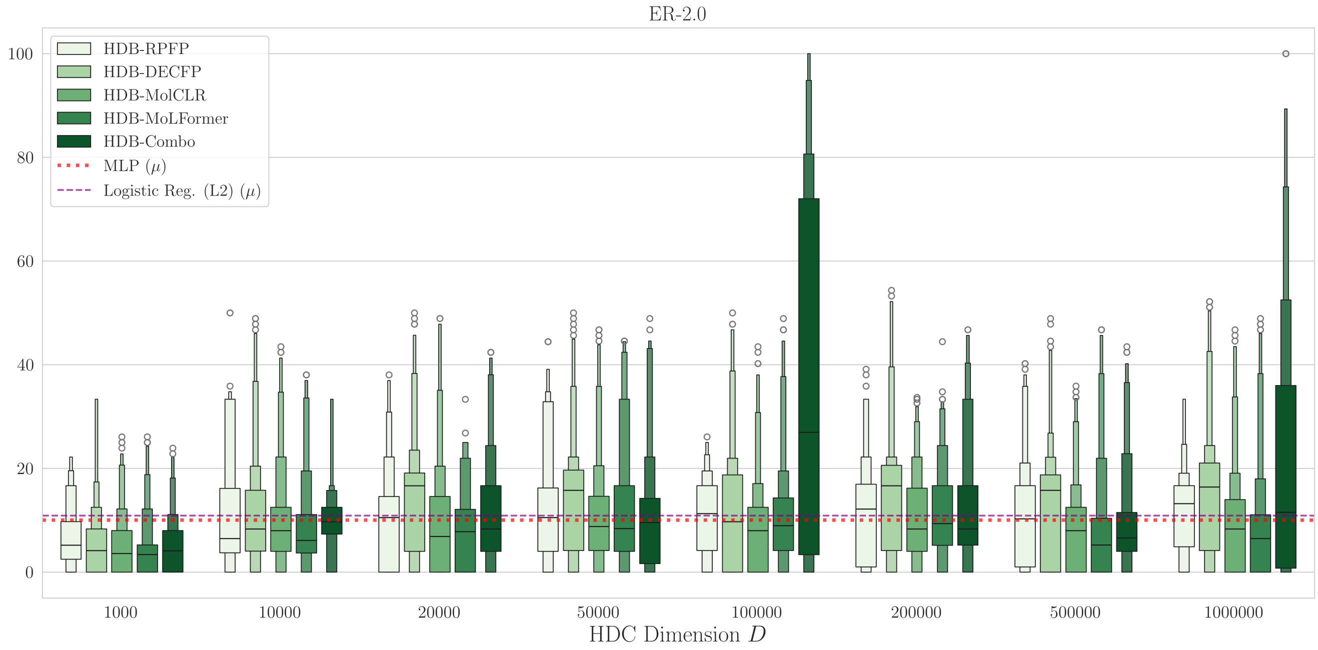

Figure 14. Distribution of the roc-enrichment with FPR=2/100, compared to MLP and logistic regression baseline models.

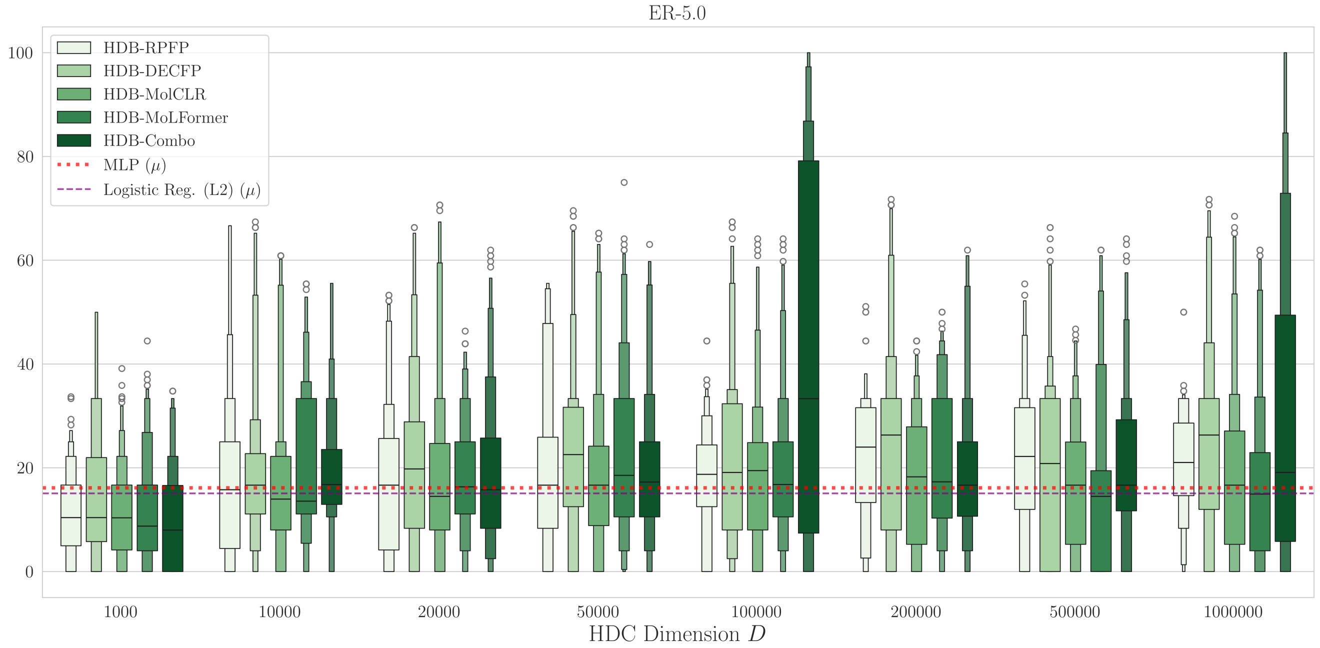

Figure 15. Distribution of the roc-enrichment with FPR=5/100, compared to MLP and logistic regression baseline models.

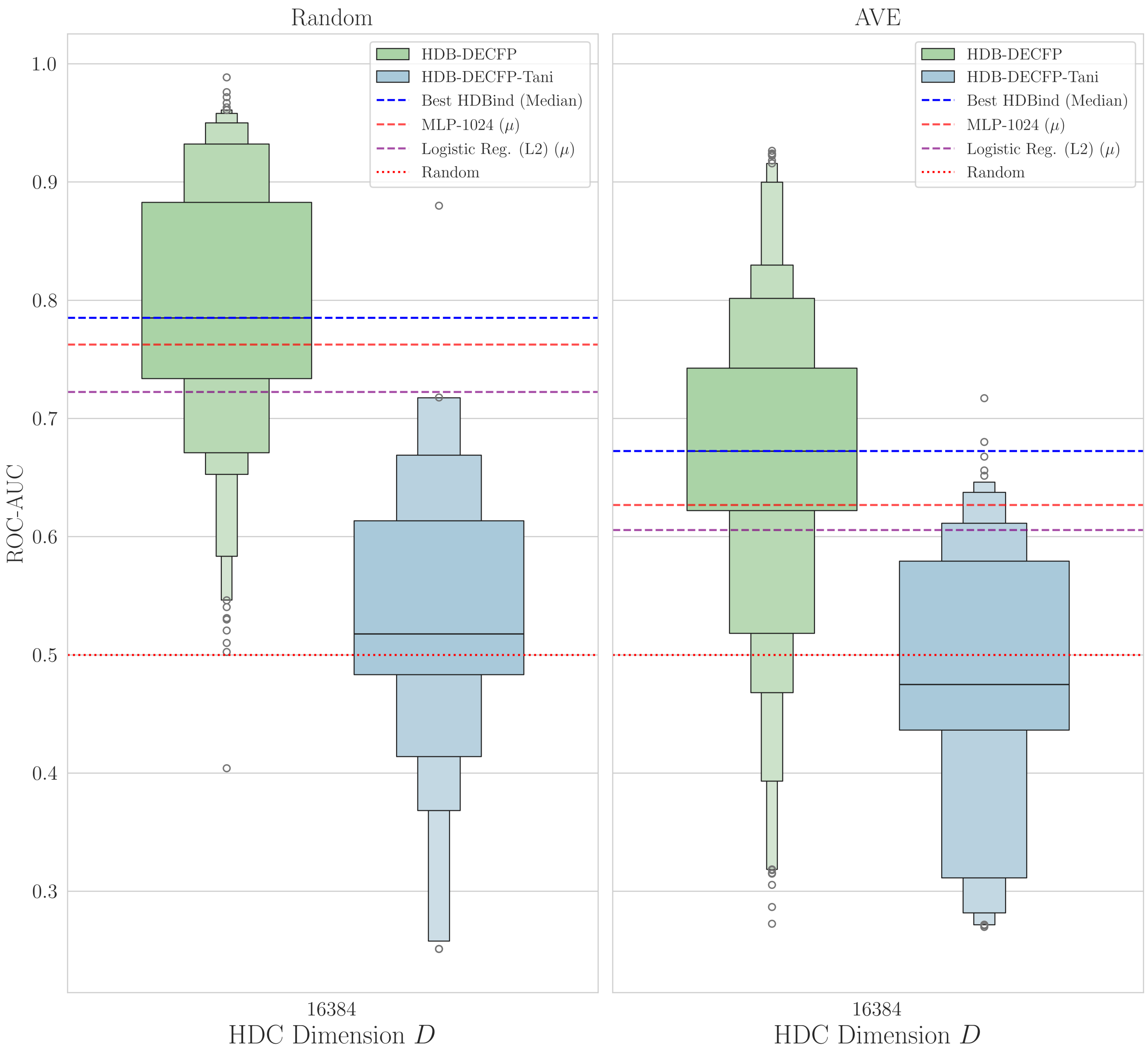

Figure 16. Distribution of roc-auc scores for HDB-DECFP models using cosine versus tanimoto similarity for training and testing with D=16,384 for each model. Our results suggest that the cosine similarity (green) consistently outperforms tanimoto similarity (blue) on both random and AVE bias-minimizing splits of the LIT-PCBA dataset.

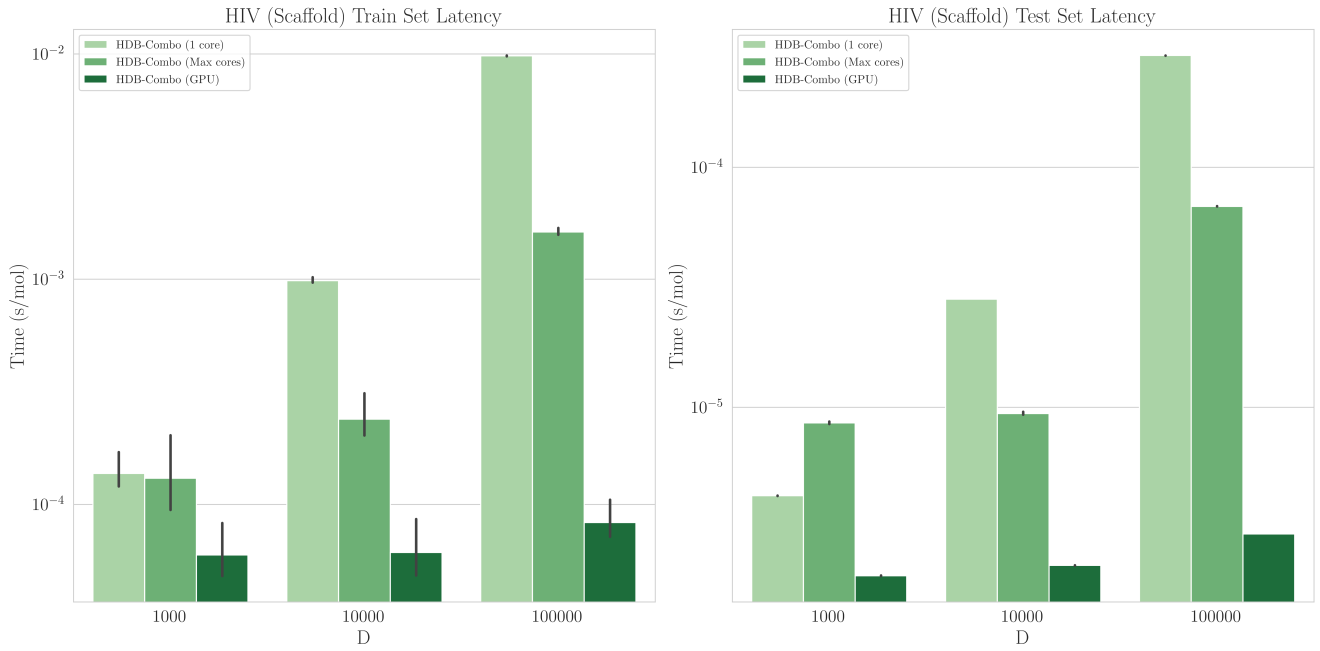

Figure 17. Comparison of the HDB-Combo training and testing latency across number of CPU cores (single or max physical cores) and GPU (with single CPU core). The HIV dataset from MoleculeNet is used. Timings are given in terms of seconds per molecule. We additionally consider the hypervector dimension size D with values of 1k, 10k, and 100k. We compare these results to the MLP baseline model using a single CPU core, max physical cores, and the GPU with a single CPU core. For each column, the leftmost distribution represents single CPU core training/testing, the middle represents max physical CPU cores, and the rightmost represents the respective GPU timings. We manually set the number of threads used by the PyTorch backend by setting the environment variable `OMP_NUM_THREADS`.

References

1. Stepniewska-Dziubinska, M. M., Zielenkiewicz, P. & Siedlecki, P. Development and evaluation of a deep learning model for protein-ligand binding affinity prediction. *Bioinformatics* **34**, 3666–3674 (2018).

2. Tran-Nguyen, V.-K., Bret, G. & Rognan, D. True accuracy of fast scoring functions to predict High-Throughput screening data from docking poses: The simpler the better. *J. Chem. Inf. Model.* **61**, 2788–2797 (2021).

3. Desaphy, J., Raimbaud, E., Ducrot, P. & Rognan, D. Encoding protein-ligand interaction patterns in fingerprints and graphs. *J. Chem. Inf. Model.* **53**, 623–637 (2013).

4. Tran-Nguyen, V.-K., Jacquemard, C. & Rognan, D. LIT-PCBA: An unbiased data set for machine learning and virtual screening. *J. Chem. Inf. Model.* (2020).

5. Rogers, D. & Hahn, M. Extended-connectivity fingerprints. *J. Chem. Inf. Model.* **50**, 742–754 (2010).

6. Ross, J. *et al.* Large-scale chemical language representations capture molecular structure and properties. *Nat. Mach. Intell.* **4**, 1256–1264 (2022).

7. Wang, Y., Wang, J., Cao, Z. & Barati Farimani, A. Molecular contrastive learning of representations via graph neural networks. *Nat. Mach. Intell.* **4**, 279–287 (2022).

8. Variorum: Vendor-agnostic computing power management.
